# Ascending aorta flow derangement is a marker of outflow obstruction in hypertrophic cardiomyopathy

**DOI:** 10.1186/1532-429X-16-S1-P293

**Published:** 2014-01-16

**Authors:** Bradley D Allen, Lubna Choudhury, Alex J Barker, Pim van Ooij, Jeremy D Collins, Robert O Bonow, James C Carr, Michael Markl

**Affiliations:** 1 Radiology, Northwestern University, Chicago, Illinois, USA; 2 Medicine - Cardiology, Norhtwestern University, Chicago, Illinois, USA; 3 Biomedical Engineering, Norhtwestern University, Chicago, Illinois, USA

## Background

Alterations in systolic blood flow are a characteristic finding in patients with obstructive hypertrophic cardiomyopathy (HCM). Echocardiography is the gold standard for hemodynamic assessment in HCM, but this technique is generally limited to measurement of the left ventricular outflow tract (LVOT) pressure gradient and may not provide a complete description of the hemodynamic impact of the disease. In this study, we sought to employ time-resolved, three-dimensional phase contrast (4D flow) MRI to visualize and quantify 3D blood flow patterns in the LVOT and ascending aorta (AAo) in patients with obstructive and non-obstructive HCM.

## Methods

**Figure 1:**
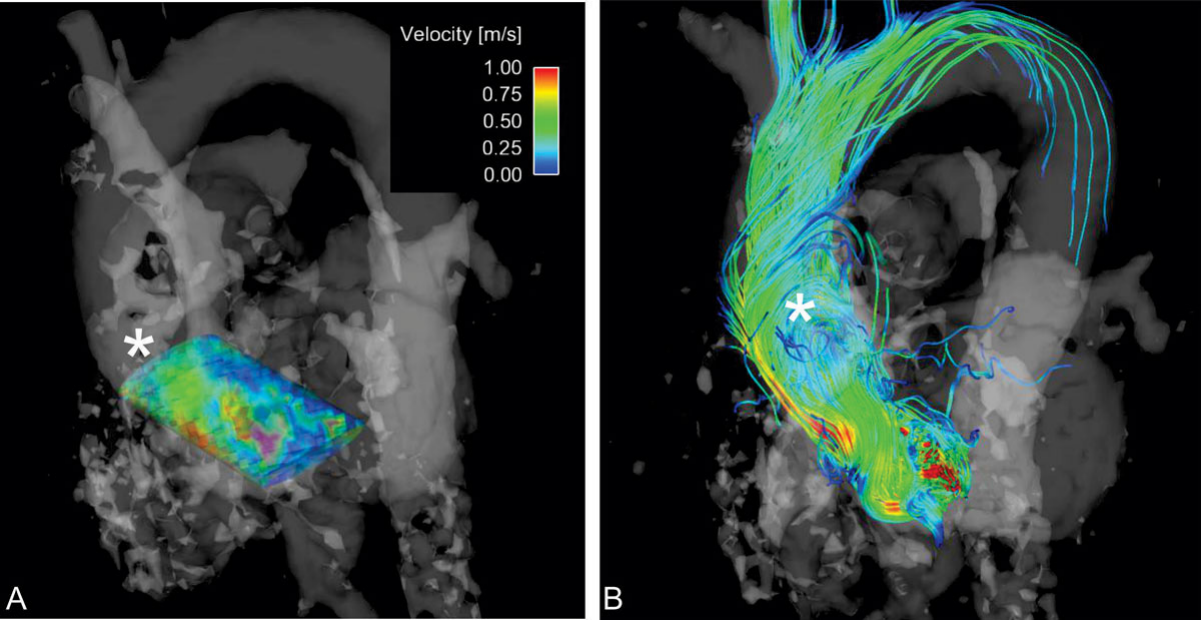
**4D flow MRI findings in a patient with obstructive HCM**. A) 3D velocity profile in the LVOT at peak systole. This volume was queried throughout the cardiac cycle to capture the peak velocity that was used in the calculation of the MRI-measured outflow tract gradient. B) Streamline representation of flow derangement in the ascending aorta (*). This patient had an average helix grade of 1.5.

Obstructive (n = 12) and non-obstructive (n = 18) HCM patients as well as 10 normal volunteers were included in this IRB-approved study. Obstruction was defined as LVOT pressure gradient > 30 mmHg on patients' most recent echocardiography study (ΔP_echo_). Septal thickness, LVOT diameter and septum/free wall ratio were measured on SSFP cine MRI. 4D flow MRI data analysis included correction for eddy currents and velocity aliasing, followed by flow visualization and quantification in dedicated software (EnSight, CEI, Apex, NC). 3D blood flow patterns within the LVOT and AAo were graded for the presence of helical flow (absent = 0, mild/moderate = 1, severe = 2) by two observers blinded to diagnosis, and the results were averaged. MRI-measured pressure gradient (ΔP_MRI_) was calculated from the peak systolic 3D blood velocity profile within the LVOT using the simplified Bernoulli equation. (Figure 1) The Mann-Whitney U test was used to compare groups and Spearman's (r_S_) or Pearson's (r) correlations were used as appropriate.

## Results

**Figure 2:**
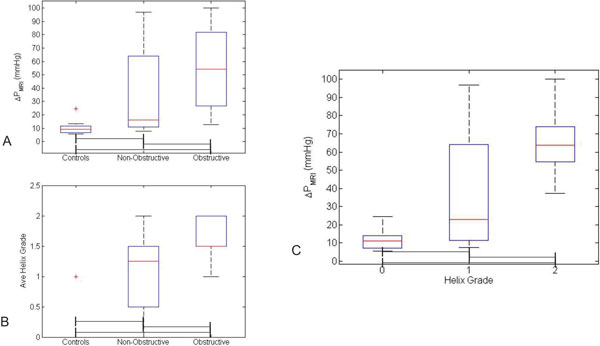
**A and B) Box plots showing the distributions of MRI-measured gradient (ΔP_MRI_) (A) and average helix grade (B) in the study cohort**. In these plots, the correlation of diagnosis with increasing gradient and helix grade is clearly displayed. C) Box plot demonstrating the distribution of ΔP_MRI_at each level of helix grade for the entire cohort. Again, a clear trend of increasing helix grade with increasing gradient is observed. Significant differences between groups (p < 0.05) are demonstrated by brackets below boxes.

There was higher grade helical flow in obstructive patients (1.6 ± 0.4) compared to both non-obstructive patients (1.1 ± 0.64, p = 0.04) and controls (0.1 ± 0.31, p < 0.001). Similarly, obstructive patients had higher ΔP_MRI_(53.8 ± 29.2 mmHg) than non-obstructive patients (33.6 ± 28.6 mmHg, p = 0.048) and controls (10.5 ± 5.6 mmHg, p < 0.001) Non-obstructive patients had higher grade helical flow (p < 0.001) and ΔP_MRI_(p = 0.004) than controls (Figure 2). In the cohort of patients, helical flow correlated with ΔP_MRI_(r_S _= 0.58, p = 0.001) and ΔP_echo _(r_S _= 0.46, p = 0.01). A significant correlations was also found between ΔP_echo _and ΔP_MRI_(r = 0.41, p = 0.03). Interestingly, ΔP_MRI _tended to be greater than ΔP_echo _(mean difference: 10.6 ± 35.3 mmHg). There were no correlations observed between helix grade or ΔP_MRI_with septal thickness, average outflow diameter, or septum/free wall ratio.

## Conclusions

Our results demonstrate that AAo flow derangement assessed using 4D flow MRI is more severe in obstructive HCM than non-obstructive HCM, and is strongly correlated with LVOT pressure gradient. This finding suggests that flow derangement is a unique marker of disease severity in this population. Further studies are required to evaluate how helical flow correlates with patient symptoms and outcomes in HCM.

## Funding

NIH NCI 5R25CA132822-04, NIH NHLBI R01HL115828; AHA13SDG14360004.

